# The Role of Intratumor Microbiomes in Cervical Cancer Metastasis

**DOI:** 10.3390/cancers15020509

**Published:** 2023-01-13

**Authors:** Lu Jiang, Baofeng Duan, Peng Jia, Yan Zhang, Xin Yan

**Affiliations:** Department of Obstetrics and Gynecology, Peking University First Hospital, Beijing 100034, China

**Keywords:** cervical cancer, metastasis, microbiome, machine learning, nomogram, ferroptosis

## Abstract

**Simple Summary:**

Microbiomes are thought to be an essential characteristic of tumors, influencing their development and progression. We found and validated certain microbiomes associated with tumor metastasis in cervical cancer samples. Furthermore, we attempted to elucidate the mechanism of the interaction between microbiomes and host cells utilizing a multiomics study. Finally, we developed an excellent prognostic prediction model for cervical cancer employing these microbiomes and their linked differentially expressed genes. This study conducted novel research concerning the link between tumor microbiomes and the host, highlighting the role of microbiomes in cervical cancer metastasis.

**Abstract:**

Background: Intratumor microbiomes can influence tumorigenesis and progression. The relationship between intratumor microbiomes and cervical cancer metastasis, however, remains unclear. Methods: We examined 294 cervical cancer samples together with information on microbial expression, identified metastasis-associated microbiomes, and used machine learning methods to validate their predictive ability on tumor metastasis. The tumors were subsequently typed based on differences in microbial expression. Differentially expressed genes in different tumor types were combined to construct a tumor-prognostic risk score model and a multiparameter nomogram model. In addition, we performed a functional enrichment analysis of differentially expressed genes to infer the mechanism of action between microbiomes and tumor cells. Results: Based on the 15 differentially expressed microbiomes, machine learning models were able to correctly predict the risk of cervical cancer metastasis. In addition, both the risk score and the nomogram model accurately predicted tumor prognosis. Differences in the expression of endogenous genes in tumors can influence the distribution of the intracellular microbiomes. Conclusions: Intratumoral microbiomes in cervical cancer are associated with tumor metastasis and influence disease prognosis. A change in gene expression within tumor cells is responsible for differences in the microbial populations within the tumor.

## 1. Introduction

In terms of incidence and mortality, cervical cancer ranks as the third most common malignancy of the female reproductive system [1]. Persistent infection with high-risk human papillomaviruses (HPV) is closely associated with the occurrence and progression of cervical cancer [2]. In most cases, cervical cancer can spread extensively through the lymphatic vessels. Once lymph nodes or distant organs are involved, the prognosis worsens [3]. According to a growing body of research, the cervicovaginal microbiome plays a significant role in the persistence, recurrence, and progression of HPV infection [4]. Nevertheless, previous studies have focused primarily on the impact of the intravaginal microbiome on HPV infection and cervical cancer pathogenesis, neglecting the role of the intratumor microbiome in tumor development and prognosis, and in particular the relationship between distant metastasis and the microbiome within cervical cancer tumors [5,6].

Tumor microbiome refers to the genome of microorganisms (bacteria, archaea, fungi, and viruses) present in the tumor parenchyma and the microenvironment surrounding the tumor [7]. It has been demonstrated that intratumor microbiomes are more often parasitized by tumor cells and immune cells within the tumor microenvironment [8,9]. Using electron microscopy, Fu et al. [10] found that approximately 3% of tumor cells contained bacteria, and approximately 97.25% of the bacteria were intracellular parasites, which could promote lung metastasis of breast cancer cells. It is believed that the tumor microbiome is one of the most important characteristics of tumors, and is present in a wide range of solid tumors and influences tumorigenesis and progression by promoting host genomic mutations and immune modulation [11]. 

As tumor tissue is a low microbial abundance environment, the identification of microbiomes is a primary concern when studying the intratumor microbiome. Over 11,000 cancer cases were catalogued for the Cancer Genome Atlas (TCGA), which is a huge, comprehensive database of cancer molecular information [12]. A workflow developed by Poore et al. [13] enables corrected microbial abundances to be derived from high-throughput sequencing data of human cancer cells, and this method was applied to create a dataset of pancancerous tumor microbial abundances derived from whole genome or RNA sequencing data for the TCGA cohort. We identified the microbiomes associated with tumor metastasis using data from multiple genomics, systematically evaluated the connection between microbiomes and host gene expression, and demonstrated the significant potential of microbiomes in the diagnosis, pathogenesis, and prognosis of cervical cancer metastasis. 

## 2. Materials and Methods

### 2.1. Preparation of Data

Microbiome data, mRNA-seq data, and clinical information for cervical cancer samples were downloaded from the TCGA–CESC cohort (https://portal.gdc.cancer.gov/; accessed on 1 November 2022). In particular, Poore et al. [13] derived microbiome data from secondary analyses of sequencing data. With regard to the total sequencing read length, approximately 7.2% of sequences of nonhuman origin were identified, of which 35.2% were identified as bacterial, viral, or archaeal, and annotated to genus-level operational taxonomic units (OTUs) by Kraken. A log2 counts per million (CPM) expression matrix was created using Voom normalization and SNM correction, available from CBioPortal (https://cbioportal-datahub.s3.amazonaws.com/cesc_tcga_pan_can_atlas_2018.tar.gz; accessed on 1 November 2022). The American Joint Committee on Cancer (AJCC) and the International Federation of Gynecology and Obstetrics (FIGO) staging systems are commonly used for staging cervical cancer. The patients were categorized according to their age, AJCC stage, and FIGO stage as well as other clinical characteristics. A total of 306 primary tumor samples were collected from the TCGA cervical squamous cell carcinoma and endocervical adenocarcinoma (CESC) cohort, of which 294 contained microbiological information. We classified patients into metastatic and nonmetastatic groups according to their N and M stages. N staging represents lymph node involvement, with N0 representing negative lymph nodes and N1 representing pelvic lymph node metastases. M staging represents distant metastases, with M0 representing non-distant metastases and M1 representing distant metastases [14]. Accordingly, patients who met both N0 and M0 were classified as nonmetastatic, and those who met N1 or M1 (any T stage) were classified as metastatic. 

Additionally, the GSE52903 dataset in the Gene Expression Omnibus (GEO) database (https://ncbi.nlm.nih.gov/geo/query/acc.cgi?acc=GSE52903; accessed on 15 November 2022) was downloaded in order to obtain gene expression profiles and clinical information for all tumor samples as an external validation dataset. This comprised 72 samples, 17 of which were normal controls and 55 of which were tumor samples [15].

### 2.2. Identification of Tumor Metastasis-Associated Microbiomes and Evaluation of Machine Learning Classification Models

Firstly, the low-abundance microorganisms (less than two samples with expression log CPM > 0) were removed, and the limma package was then applied between the progressive and nonprogressive tumor groups to identify microbiomes at the genus level that were differentially expressed in the two groups based on screening criteria of log2 fold change (FC) ≥ 0.4 and *p* value < 0.05. Differentially expressed microbiomes were utilized as an explanatory variable, and cervical cancer metastases were chosen as the response. After dividing all 142 samples into training and test sets, three machine learning models were built and tested using the caret and DALEX packages to plot cumulative residual curves using random forest, support vector machine, and generalized linear models. The Mleval package was used to plot ROC curves for the tenfold cross-validation case to select the best training model. The importance of variables was calculated using the wrapper method, and the best combination of variables was filtered using recursive feature elimination (RFE) [16].

### 2.3. Construction and Evaluation of Nomogram 

After obtaining the optimal combination of variables, we created a nomogram model using the rms package to calculate cervical cancer metastasis risk scores based on the sum of projections of each variable and corresponding to the likelihood of metastases. Calibration curves, decision curve analysis (DCA), and clinical impact curves were employed to assess the reliability of the model.

### 2.4. Tumor Typing Based on Metastasis-Associated Microbiomes

Consequently, 294 samples with survival-related information were retained. In order to define differentially expressed microbiomes (DEMs) associated with survival, univariate Cox regression analysis was performed on all screened metastasis-associated microbiomes using the survival package. The ConsensusClusterPlus package was used to cluster all 294 samples, which were consistently clustered into two tumor subtypes. On the basis of overall survival time and status, Kaplan–Meier curves were used to compare survival differences between the two groups.

### 2.5. Immune Infiltration of Tumors with Different DEM Clusters

CIBERSORT is a deconvolution algorithm that enables the calculation of the proportion of immune cell types in a given tissue by combining the genomes of marker cells from different immune cell subpopulations [17]. We calculated immune cell infiltration in tumors of different DEM clusters using the CIBERSORT website (https://cibersortx.stanford.edu; accessed on 15 November 2022) and compared the expression levels of common immune checkpoints across different clusters.

### 2.6. Gene Expression Analysis and Functional Enrichment of Tumors with Different DEM Clusters

Differentially expressed genes (DEGs) of different DEM clusters were screened based on the RNA-seq expression of 297 samples using the limma package with the screening criteria of |log2 fold change (FC)| ≥ 0.5 and *p* value < 0.05. On the basis of these DEGs, Gene Ontology (GO) and Kyoto Encyclopedia of Genes and Genomes (KEGG) analyses were performed using the clusterProfiler package.

### 2.7. Development and Validation of a Risk Scoring System Based on DEM and DEG

We divided all 294 samples into training and test sets and carried out univariate Cox regression analysis and LASSO regression screening variables utilizing all screened DEMs and DEGs as risk factors. We then used a multivariate Cox regression model with the STEP method for iteration, producing an AIC (Akaike information criterion) minimum multivariate Cox regression model with risk score = $\overset{n}{\underset{i=1}{\sum }}expression\;of\;risk\;factor\;i\times coefficient$. To assess the predictive validity of the risk score model, we compared the risk scores of different DEM clusters in the training and test sets, and we used the survivalROC package to plot time-dependent ROC curves of risk scores on overall survival (OS) at 1, 3, and 5 years. In addition, Kaplan-Meier curves were plotted by dividing all populations into high- and low-risk groups in accordance with their risk scores. As an external validation set, we calculated individual risk scores for samples from the GSE52903 dataset in order to determine the predictive validity of the risk scores.

### 2.8. Validation and Development of a Prognostic Nomogram for Cervical Cancer

Traditionally, cervical cancer prognosis is determined by pathological stage and type. In order to predict the 1-year, 3-year, and 5-year OS rates of patients, we added risk scores to FIGO stage, AJCC stage, and age to create a nomogram model of cervical cancer prognosis. To verify the reliability of the nomogram model, we plotted calibration, time-dependent ROC, and DCA curves.

### 2.9. The Relationship between Risk Score and Immune Infiltration

Based on the results of the CIBERSORT algorithm, we assessed the correlation between risk score and immune cell abundance. In addition, the ESTIMATE algorithm was used to determine the stromal, immune, and ESTIMATE scores in each sample, which were compared between the high- and low-risk groups [18]. A heatmap was produced to show the correlation between the expression levels of independent risk factors in the risk assessment model and the abundance of 22 immune cells.

### 2.10. Statistical Analysis

Data processing, analysis, and presentation were carried out using R software (version 4.1.2) and its relevant packages. Sangerbox (http://www.sangerbox.com/tool, accessed on 2 January 2023) and ImageGP (http://www.ehbio.com/ImageGP/, accessed on 2 January 2023) were also used to visualize the results [19,20]. A two-sided *p* value of 0.05 was considered significant.

## 3. Results

### 3.1. Identification of Microbiomes Associated with Cervical Cancer Metastasis

**Figure 1:**
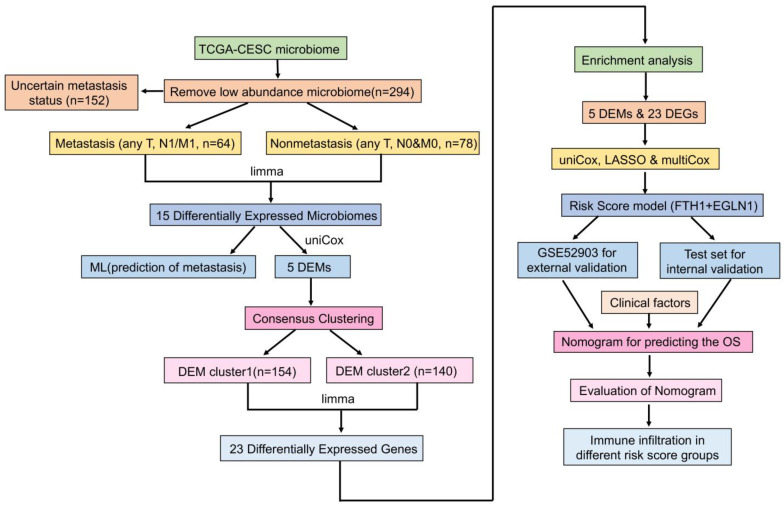
Flow chart illustrating the analysis of tumor metastasis-associated microbiomes in the TCGA–CESC.

**Figure 2:**
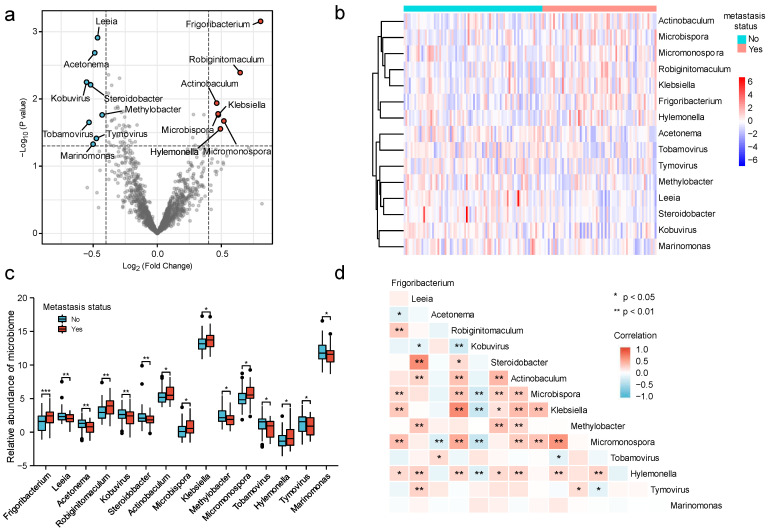
Identification and comparison of microbiomes associated with cervical cancer metastasis. (**a**) Volcano plot of differentially expressed microbiomes, with red dots indicating high expression in the metastasis group, and blue dots indicating high expression in the non-metastasis group; (**b**) heatmap showing the relative abundance of 15 microbiomes in each sample; (**c**) box plots comparing the relative abundance of 15 microbiomes between the metastasis and non-metastasis groups; and (**d**) analysis of Spearman’s correlation between 15 microbiomes. (*p* < 0.05 *; *p* < 0.01 **; *p* < 0.001 ***).

Figure 1 illustrates the flow chart of this study. First, we classified all the samples according to metastasis status, and then obtained the differentially expressed microbiome. Next, we evaluated the predictive value of these microbiomes for metastasis and constructed a microbial classification system for tumors. Through the systematic analysis of tumors with different microbial classifications, we screened the prognosis-related risk factors, and finally built a fairly reliable prognosis prediction model. There were a total of 294 samples with microbial information, 64 of which were classified as metastasis groups, and 78 as non-metastasis groups, and clinical information for each group is presented in Table 1. There were 1406 microbiomes classified at the genus level, which comprised 62 archaea, 138 viruses, and 1206 bacteria. Of these, 1396 microorganisms remained after the low-abundance microbiomes were removed. Of the 15 differentially expressed microbiomes screened, 7 and 8 were highly expressed in the metastasis and non-metastasis groups, respectively. Figure 2a is a volcano plot showing the distribution of differentially expressed microbiomes, and Figure 2b, c illustrates the relative abundance of the 15 differentially expressed microbiomes. Based on Spearman correlation analysis, Figure 2d shows that *Klebsiella* and *Robiginitomaculum* had the highest positive correlation, whereas *Micromonospora* and *Kobuvirus* had the highest negative correlation.

### 3.2. Model Construction and Feature Selection in Machine Learning

**Figure 3:**
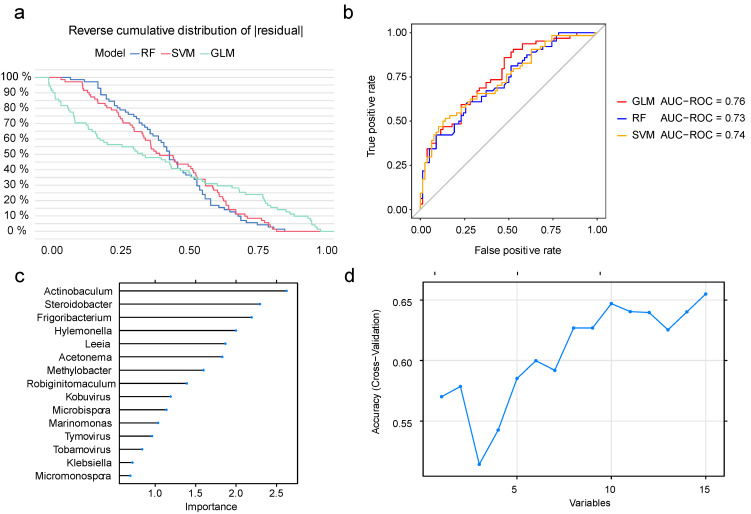
Model construction and feature screening using machine learning algorithms. (**a**) Diagram of the reverse cumulative distribution of residuals in the RF, GLM, and SVM models; (**b**) ROC curves and AUC values for the three models; (**c**) ranking of feature importance; and (**d**) feature–accuracy curves for generalized linear models. RF, random forest; GLM, generalized linear model; SVM, support vector machine.

The ability of the screened metastasis-associated microbiomes to predict cervical cancer metastasis was tested using three machine learning models: random forest (RF), generalized linear model (GLM), and support vector machine (SVM). According to the cumulative residual curve (Figure 3a) and the receiver operating characteristic (ROC) curve (Figure 3b), the generalized linear model was the most effective machine learning model. In Figure 3c, we ranked the importance of all the microbial features, and the feature–accuracy curve (Figure 3d) indicated that the model was most accurate when all 15 microbial features were included.

### 3.3. The Nomogram Model for Prediction of Cervical Cancer Metastasis

**Figure 4:**
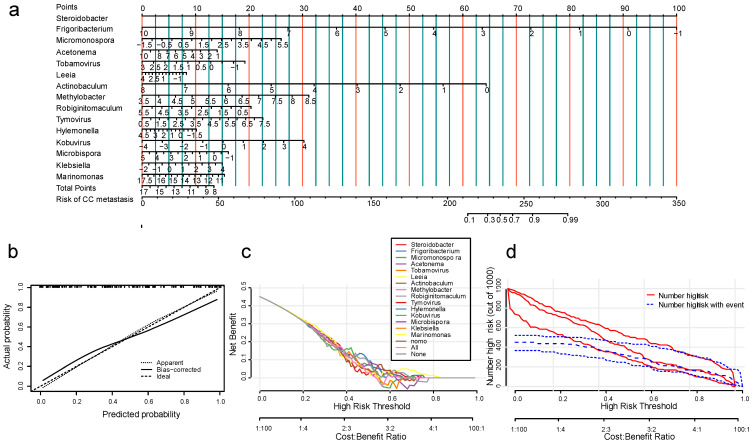
Construction and validation of nomogram models. (**a**) Nomogram model based on 15 microbial features; (**b**) calibration curve of the nomogram; (**c**) clinical decision curve (DCA) of the nomogram; and (**d**) clinical impact curve of the nomogram showing the prediction accuracy and cost-benefit ratio of the nomogram under different risk thresholds.

Machine learning models have high predictive power but poor practical utility, so we constructed a nomogram model (Figure 4a) based on the screened microbial features and then validated it. Calibration curves revealed that microbial features accurately predicted cervical cancer metastasis (Figure 4b). At certain probability thresholds, the DCA demonstrated that the nomogram model was able to achieve a higher net benefit than individual microbial features, and thus is more applicable to clinical practice (Figure 4c). The clinical impact curves illustrate the comparison of the cost–benefit ratio between predicted and true metastasis status for different risk thresholds (Figure 4d). 

### 3.4. DEM-Based Tumor Typing and Prognosis, Immune Infiltration

**Figure 5:**
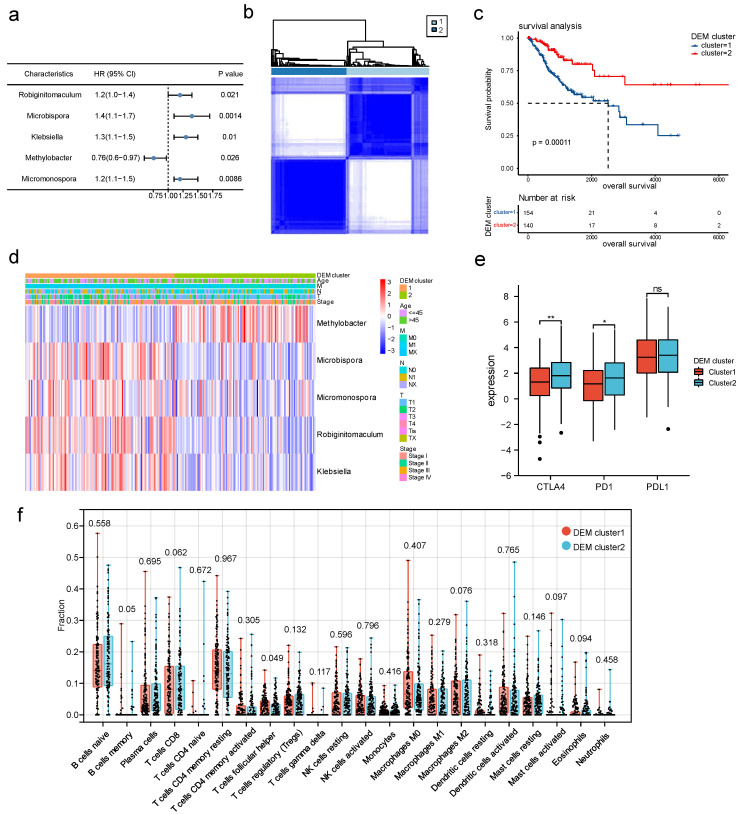
DEM-based tumor typing and prognosis, immune infiltration. (**a**) Forest plot of univariate Cox regression analysis of five microorganisms associated with survival; (**b**) consistent clustering of all cervical cancer samples into two subtypes based on DEM; (**c**) K–M survival curves of two DEM clusters; (**d**) heatmap showing the expression of five DEMs and their relationship with DEM clusters, age, FIGO stage, and TNM stage; (**e**) DEM clusters with different expressions of three immune checkpoints, PD1, PDL1, and CTLA4; and (**f**) immune cell infiltration of tumors with different DEM clusters (the number above the boxplot represents the *p* value). (*p* < 0.05 *; *p* < 0.01 **).

To classify cervical cancer into different types using microbial features, we selected the simplest possible typing method. Based on the expression of all 15 microbiomes, univariate Cox regression analysis found that five microbiomes, *Methylobacter*, *Robiginitomaculum*, *Klebsiella*, *Micromonospora,* and *Microbispora* were associated with survival. Of these, *Methylobacter* showed a negative association with mortality risk, and the other four had a positive association with mortality risk. Figure 5a shows the forest plot. We defined these five microorganisms associated with survival as differentially expressed microbiomes (DEMs), and their relative abundance was used to categorize all patients into two clusters (Figure 5b). The K–M curves indicated that DEM cluster 2 had a significantly better prognosis than DEM cluster 1 (Figure 5c). Figure 5d shows the expression of five microbiomes in different DEM clusters. DEM cluster 1 was characterized by *Robiginitomaculum*, *Microbispora*, *Klebsiella,* and *Micromonospora*, whereas DEM cluster 2 was characterized by *Methylobacter*. In DEM cluster 2, cytotoxic T-lymphocyte-associated protein 4 (CTLA-4) and programmed cell death protein 1 (PD-1) expression levels were significantly higher than in DEM cluster 1, indicating that DEM cluster 2 would be more likely to benefit from immunotherapy (Figure 5e). In addition, the immune microenvironment of the tumor differed slightly between the DEM subtypes, such as higher levels of CD8^+^ T-cell infiltration in DEM cluster 2 and Treg cell infiltration in DEM cluster 1, but none of these differences were statistically significant (Figure 5f).

### 3.5. The DEGs and Functional Enrichment Derived from the DEM Cluster

**Figure 6:**
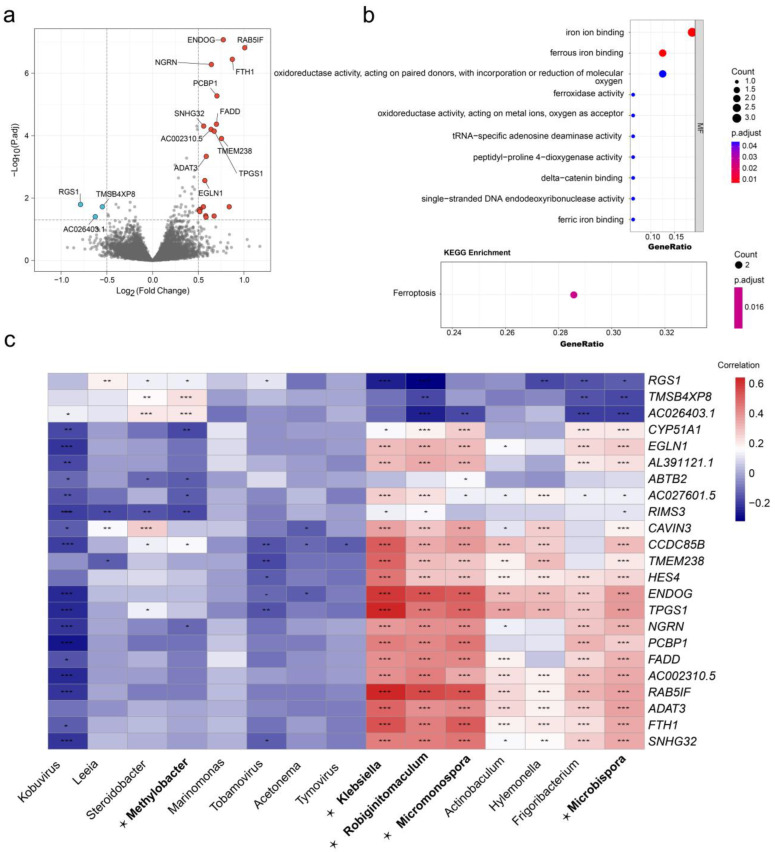
DEG and functional enrichment analysis of different DEM clusters. (**a**) Volcano plot of DEG distribution. Red dots indicate high expression in DME cluster 1, and blue dots indicate high expression in DEM cluster 2; (**b**) analysis of DEGs based on GO and KEGG functional enrichment; and (**c**) heatmap of correlation between DEGs and all differentially expressed microbiomes, microbiomes in bold and marked with ⋆ are DEMs. (*p* < 0.05 *; *p* < 0.01 **; *p* < 0.001 ***).

Differential expression analysis was conducted using mRNA-seq data to identify 23 DEGs in cervical cancer tumors with different DEM clusters. The results showed that 20 genes were highly expressed in DEM cluster 1 and 3 genes were highly expressed in DEM cluster 2 (Figure 6a). We then performed functional enrichment analysis on all 23 DEGs, and GO analysis indicated that ferritin heavy chain 1 (FTH1), egl-9 family hypoxia-inducible factor 1 (EGLN1), and cytochrome P450 family 51 subfamily A member 1 (CYP51A1) were enriched in the iron and ferrous ion-binding pathways. The KEGG analysis revealed that FTH1 and PCBP1 were enriched in the ferroptosis pathway (Figure 6b). A heatmap of the correlation between all differentially expressed genes and differently expressed microbiomes was plotted (Figure 6c).

### 3.6. The Development and Validation of a Cervical Cancer Prognostic Risk Score Model

**Figure 7:**
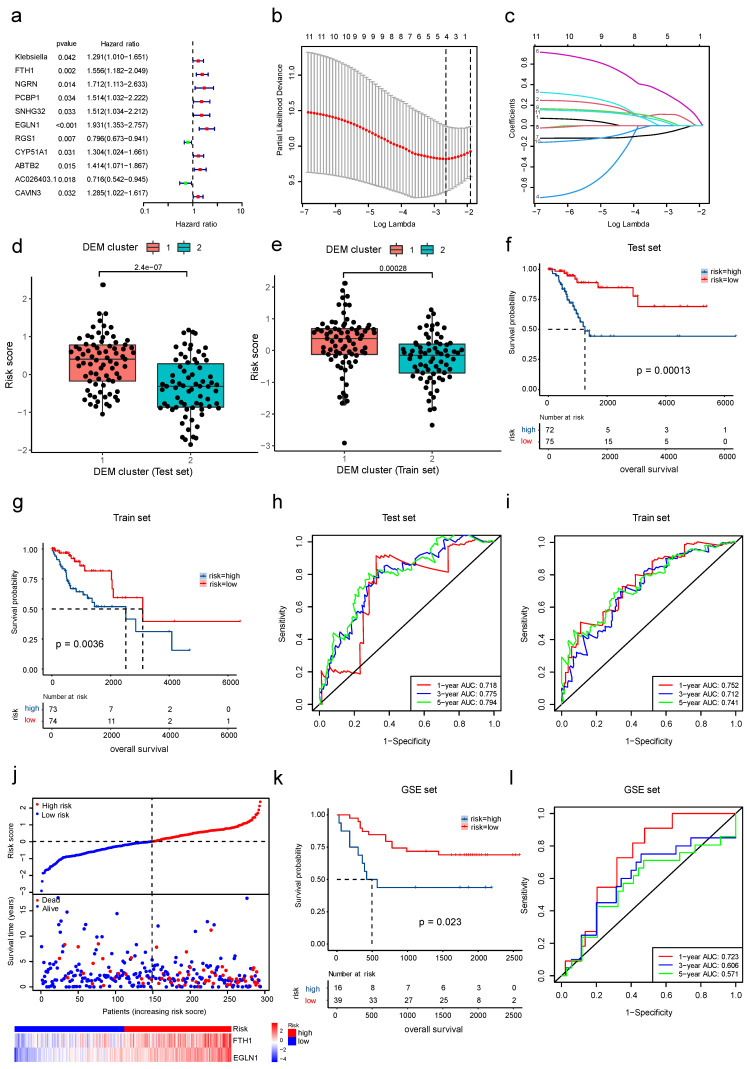
The construction and validation of a prognostic risk score model. (**a**) Univariate Cox regression in the training set to screen for survival-related variables, risk factors for survival are on the right side of the dotted line, and protective variables are on the left.; (**b**) cross-validation curves of LASSO regression; (**c**) path coefficient plots of LASSO regression; (**d**,**e**) comparison of prognostic risk scores for different DEM clusters; (**f**,**g**) K–M curves for patients with different risk score classes; (**h**,**i**) ROC curves for predictive validity of risk score models for 1-year, 3-year, and 5-year OS rates; (**j**) scatter plots showing survival status, risk score distribution, and heatmaps of expressions for two key variables for all patients; (**k**) prognostic K–M curves for patients with different risk score classes in the GSE52903 cohort; and (**l**) ROC curves for predictive validity of the risk score model in the GSE52903 cohort.

In order to construct a prognostic scoring system for cervical cancer, we used gene expressions in all DEMs and DEGs with a total of 28 variables as predictors. All patients were randomly assigned to a training set (n = 147) and a test set (n = 147) in a 1:1 ratio, and the two groups were matched according to the percentage of deaths. In the training set, the 28 variables were sequentially screened using univariate Cox regression (Figure 7a), LASSO regression (Figure 7b,c), and multivariate Cox regression analysis to determine the best model. As a result, we obtained an optimal risk score model with risk score = 0.298 × expression of FTH1 + 0.548 × expression of EGLN1, which had a C-index = 0.722.

Subsequently, by comparing the prognostic risk scores of the different DEM clusters in both the training and test sets, we found that DEM cluster 1 had a significantly higher prognostic risk score than DEM cluster 2 (Figure 7d,e). According to their risk scores, we divided all the patients into a high-risk group and a low-risk group, and the KM curve analysis revealed that the prognosis of the high-risk group was significantly worse in both training and testing (Figure 7f,g). The areas under the ROC curve for the predictive validity of the risk score model for the 1-year, 3-year, and 5-year OS rate in the test set were 0.718, 0.775, and 0.794 (Figure 7h), respectively, and the areas under the ROC curve in the training set were 0.752, 0.712, and 0.741 (Figure 7i), respectively, indicating that the risk score model was fairly accurate in predicting OS rate. The expression of the two genes included in the risk score model was plotted as a heatmap, and the risk score and survival status scatter plots showed that as the risk score increased, mortality increased, and OS rate gradually decreased (Figure 7j).

Moreover, to demonstrate the generalizability of the risk score model, we calculated risk scores for 55 tumor patients in the GSE52903 cohort. The KM curves revealed that patients with high risk had significantly worse prognoses than those with low risk (*p* = 0.023) (Figure 7k). According to the ROC curves, the areas under the AUC curve for the model’s 1-year, 3-year, and 5-year OS rate predictions were 0.723, 0.606, and 0.571, respectively (Figure 7l).

### 3.7. Nomogram Model for Cervical Cancer Prognosis and Validation

**Figure 8:**
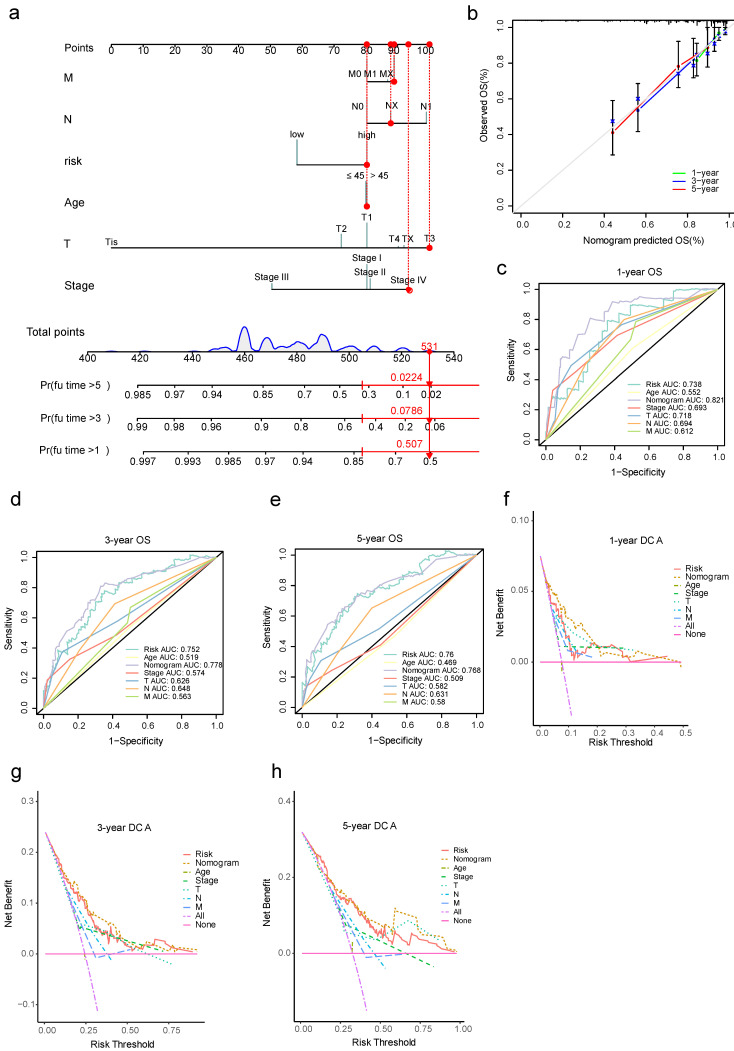
Development and validation of the nomogram model for the prognosis of cervical cancer. (**a**) Scoring system of the nomogram model, where red dots indicate the scores of each index, total score, and predicted survival probability for patient number 4; (**b**) calibration curves of the nomogram model; (**c**–**e**) ROC curves of the 1-year, 3-year, and 5-year OS rate predictive validity of the nomogram model and other independent predictors; (**f**–**h**) clinical decision curves for the 1-year, 3-year, and 5-year OS rates using the nomogram model and other independent predictors.

In order to make the risk scoring system more practical, we incorporated FIGO stage, TNM stage, age, and risk score to build a nomogram model of cervical cancer prognosis for predicting 1-year, 3-year, and 5-year OS rate (Figure 8a). To validate the model, we used patient number 4, with a FIGO stage IV, T3NXMX, age less than 45 years, and a risk score grouped as high-risk, resulting in a total score of 531, predicting a survival rate of 50.7% at 1 year, 7.86% at 3 years, and 2.24% at 5 years. Notably, the survival rate of this patient was 2.27 years, indicating the high predictive validity of this model. Calibration curves showed a good agreement between actual and model-predicted risks (Figure 8b). ROC curves were plotted separately for 1-year, 3-year, and 5-year OS rates using independent predictors and the nomogram model, and these showed that the nomogram model had greater predictive validity than the independent predictors (Figure 8c–e). As can be seen from the clinical decision curves, the nomogram model consistently achieved greater net benefits at most probability thresholds (Figure 8f–h).

### 3.8. The Relationship between Prognostic Risk Score and Immune Cell Infiltration

**Figure 9:**
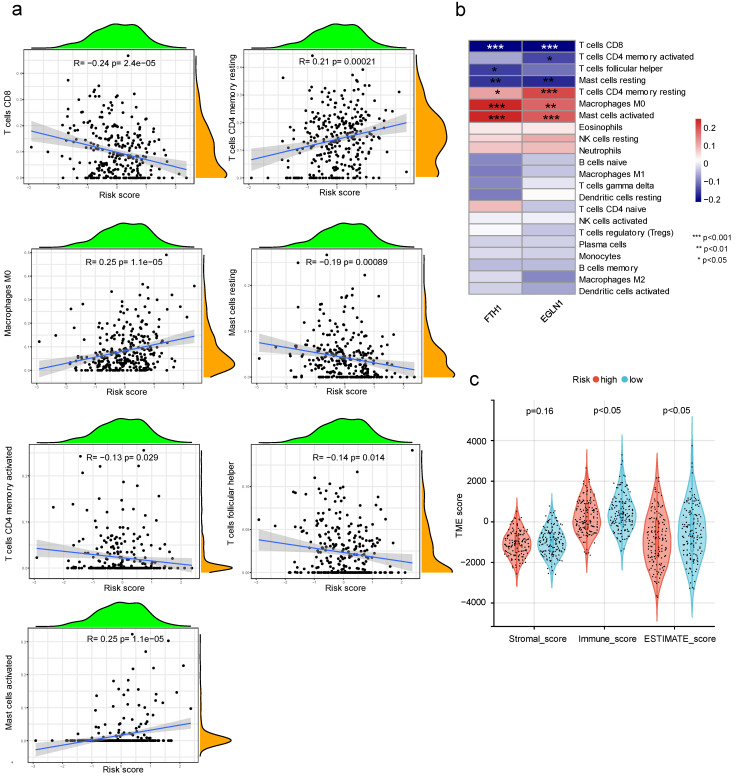
Relationship between risk score and immune infiltration. (**a**) Analysis of immune cell types associated with risk score (R represents Pearson correlation coefficient; p represents the significance levels); (**b**) heatmap of correlation between expression levels of FTH1 and EGLN1 and immune cells; and (**c**) immunity scores observed within different groups of risk scores. (*p* < 0.05 *; *p* < 0.01 **; *p* < 0.001 ***).

The expression of 22 immune cells within the tumor was determined using the CIBERSORT algorithm and correlated with the risk score. It was found that the risk score correlated positively with the infiltration of resting memory CD4^+^ T cells, M0 macrophages, and activated mast cells, and negatively with the infiltration of CD8^+^ T cells, activated CD4^+^ memory T cells, Tfh (follicular helper T cells), and resting mast cells (Figure 9a). Two key genes (FTH1 and EGLN1) were also associated with different immune cells (Figure 9b). In addition, both the Immune score and ESTIMATE score were lower in the high-risk group than in the low-risk group (Figure 9c).

## 4. Discussion

The definitive cause of cervical cancer is persistent infection with high-risk HPV, and the vaginal microbiome can have a significant impact on HPV infection and cervical precancer by altering pH levels and lactate and hydrogen peroxide concentrations in the vagina and by directly interacting with cervical epithelial cells [21,22,23]. An in vitro experiment showed that lactic acid, a metabolite of *Lactobacillus iners,* activates the Wnt pathway through the lactate-Gpr81 complex, thereby increasing the level of core fucosylation in epithelial cells and inhibiting the proliferation and migration of cervical cancer cells [24]. In contrast to open cavities such as the lower genital and gastrointestinal tracts, there is a low abundance of microorganisms in tumor tissue. However, they are still a significant component of the tumor microenvironment, and the specificity of these microbiomes within specific tumors suggests that they might be associated with tumorigenesis and progression [11]. Microbiomes may contribute to tumor development in three different ways: (1) directly promoting tumorigenesis by increasing mutations, (2) modulating oncogenes or oncogenic pathways, and (3) inhibiting or promoting tumor progression by modulating the immune system of the host [25]. Poore et al. developed a workflow that allowed us to hybridize the data in TCGA with microbiomics, in which approximately 2% of all sequences in the CESC project were identified as being of microbial origin [13]. Using the results of the above research, microbial abundance was found to be a better predictor of prognosis in cervical cancer than clinical factors [26]. Understanding the mechanism of cervical cancer metastasis can assist in identifying high-risk cervical cancer cases early and reducing their lethality. In recent years, research on the mechanisms of cervical cancer metastasis has focused on oncogenes and their associated signaling pathways, and some recent studies have also demonstrated the regulatory role of noncoding RNAs [27]. Fu et al. [10] found that intracellular bacteria could promote lung metastasis of breast cancer cells, the mechanism of which may be that intracellular bacteria invade tumor cells, remodel the cytoskeleton via the RhoA–ROCK pathway, enhance the tolerance of circulating tumor cells to intravascular mechanical pressure, and reduce cell death during metastasis. If microbes are involved in the metastasis of breast cancer, could the same be true of the microbiomes within cervical tumors?

According to our findings, some intratumor microbiomes associated with cervical cancer can effectively predict metastases and are closely related to the prognosis of cervical cancer. Among them, we identified 15 genera of microbiomes as being associated with cervical cancer metastasis. A machine learning model and nomogram model of these 15 microbiomes were capable of accurately predicting the risk of cervical cancer metastasis. Overall survival rate was also positively correlated with five of these microbiomes. The risk of death from cervical cancer was positively associated with *Robiginitomaculum*, *Klebsiella*, *Micromonospora*, and *Microbispora*, but negatively associated with *Methylobacter*. It is possible to predict the prognosis of cervical cancer by evaluating the relative abundance of these five microbiomes and the corresponding tumor classification. *Klebsiella* has been linked to the incidence and development of a variety of malignancies. An in vitro investigation demonstrated that *Klebsiella pneumoniae* might increase the generation of reactive oxygen species and the expression of HIF-mRNA in airway epithelial cells, resulting in epithelial mesenchymal transition, which is frequently the foundation of tumor cell metastasis [28]. In clinical observation, the prevalence of *Klebsiella* was much higher in esophageal squamous cell carcinoma tissues than in healthy esophageal tissues, and *K. pneumoniae* in the bile of patients with pancreatic ductal adenocarcinoma can lead to gemcitabine resistance and worsen prognosis [29,30]. Furthermore, *Klebsiella* may also be involved in the development of bladder and colorectal cancers [31,32]. The other four bacteria are not abundant in cervical cancer tissue, and few reports have been published that relate it to human disease.

However, do these low-abundance microbes enter the tumor or immune cells at random? Do tumor cells play an active selection role in this process? This area has rarely been explored in previous studies. By combining transcriptomic and microbiomic analyses of tumors, our study demonstrated that microbial signatures predisposed to tumor metastasis were also associated with the gene expression profiles of tumor cells. Specifically, tumors with high expression of genes such as FTH1, EGLN1, and BCPB1 had more microbiomes associated with tumor metastasis. Most of these genes were enriched in pathways such as ferroptosis and iron transport. Ferroptosis is an iron-dependent programmed cell death that disrupts the structural integrity of cell membranes through the accumulation of lipid peroxides, which in turn leads to cell death [33]. As tumor cells are metabolically active and produce large amounts of reactive oxygen species (ROS), they require more iron reserves to maintain high levels of iron death, and iron depletion inhibits tumor cell growth and metastasis through this mechanism [33,34]. FTH1 encodes the ferritin heavy chain, a key subunit of ferritin that plays an important role in catalyzing the Fe^2+^ oxidation reaction, and FTH1 overexpression can lead to iron overload and inhibit ferroptosis [35]. An in vitro experiment demonstrated a significant increase in ferrous iron levels and ROS levels in macrophages after infection with bacteria, and the transportation of iron into bacterial vesicles was also found to induce bacterial death, which may be an effective defense mechanism for cellular clearance of pathogen infection [36]. Taking the results of our study into account, altered endogenous ferroptosis regulators may decrease bacterial clearance by inhibiting ferroptosis. This, in turn, allows bacteria to survive in these cells and perform pro-tumorigenic and metastatic functions. The upregulation of FTH1 in tumor cells or immune cells, for example, may contribute to a reduction in the clearance of bacteria by inhibiting ferroptosis. 

Based on differentially expressed genes from different DEM clusters, we constructed a risk score model for cervical cancer prognosis and tested the predictive value of the model. Based on the results, the model had high predictive power for the 1-year, 3-year, and 5-year cervical cancer OS rates and could be extrapolated to the GSE dataset for validation. In addition to being associated with prognosis, the risk score was also related to the mode of immune infiltration. We found a reduced abundance of cells with antitumor immunity, such as CD8^+^ T cells and Tfh cells, together with a lower immune score and ESTIMATE score in the high-risk group, suggesting that higher tumor purity and absence of immune cells is another high-risk factor for tumor metastasis [18,37].

In this study, we propose a potential novel mechanism of interaction between tumor progression- and metastasis-associated microbiomes and host cells; however, some limitations are involved. Firstly, only primary tumors were sampled, and samples from metastatic lesions were not available for examination of the enrichment of intratumor microbiomes. Secondly, although the scoring system was validated using an external cohort, it was still derived from a public database and requires additional data with larger sample sizes in order to be prospectively validated. In addition, the data in the microbial database are normalized gene expressions, which can only provide information about the relative abundance of microorganisms within the tumor tissue rather than the absolute number, which is a prerequisite for understanding the role of microorganisms in cancer cells. As a final point, further in vivo and ex vivo experiments are required to determine the mechanism of action of tumor cells and microbiomes. There are still outstanding questions in the field of tumor microbiomes, such as, by what mechanisms microbiomes bind to and invade tumor/immune cells, whether tumor/immune cells passively invade or actively recruit microbiomes to acquire novel capabilities, and what other roles microbiomes within tumor cells play in the process of tumor metastasis. The answers to these questions will help us to find new targets for tumor treatment.

## 5. Conclusions

By comparing the gene sequences of microbial origin in the TCGA–CESC database with different metastatic status, we obtained 15 differentially expressed microbiomes, which we then used to construct machine learning and nomogram models to predict the risk of cervical cancer metastasis. Five of these microbiomes (*Robiginitomaculum*, *Klebsiella*, *Micromonospora*, *Microbispora*, and *Methylobacter*) were associated with cervical cancer prognosis, and, dependent on their expression, we built tumor microbiome clusters. Additionally, based on differentially expressed genes in patients with different DEM clusters, we constructed a model for prognostic risk scoring of cervical cancer patients, which achieved accurate prediction in the training set, test set, and external cohort. Lastly, we hypothesized that the differential expression of endogenous genes in tumor tissues could influence the type and distribution of intracellular microbiomes through functional enrichment analysis. For example, upregulation of the FTH1 gene inhibits the destructive effect of ferroptosis on intracellular microbiomes, resulting in a microbial state that is more favorable for tumor metastasis. These findings expand the field of tumor microbial study and contribute to the identification of new targets for tumor therapy as well as to the reduction of tumor metastasis and mortality rates through the use of microbiomes and differentially expressed genes to predict tumor prognosis.

## Figures and Tables

**Table 1:** Comparison of clinical baselines for different metastasis status subgroups.

Characteristic	Non-Metastasis	Metastasis	All *
n	78	64	294
M, n (%)			
M0	78 (100%)	25 (39.1%)	111 (37.8%)
M1	0 (0%)	9 (14.1%)	9 (3.1%)
MX	0 (0%)	30 (46.8%)	174 (59.1%)
N, n (%)			
N0	78 (100%)	2 (3.1%)	129 (43.9%)
N1	0 (0%)	57 (89.1%)	57 (19.4%)
NX	0 (0%)	5 (7.8%)	108 (36.7%)
T, n (%)			
T1	56 (71.8%)	30 (46.9%)	134 (45.6%)
T2	20 (25.6%)	22 (34.4%)	71 (24.2%)
T3	2 (2.6%)	7 (10.9%)	20 (6.8%)
T4	0 (0%)	3 (4.7%)	8 (2.7%)
Tis	0 (0%)	1 (1.6%)	1 (0.3%)
TX	0 (0%)	1 (1.6%)	60 (20.4%)
age, mean ± SD	47.26 ± 12.88	45.36 ± 11.93	48.22 ± 13.91

* All refers to all samples with microbial information; Tis: tumor in situ.

## Data Availability

These data were derived from the following resources available in the public domain: (https://cbioportal-datahub.s3.amazonaws.com/; accessed on 1 November 2022) and (https://ncbi.nlm.nih.gov/geo/ query/acc.cgi?acc= GSE52903; accessed on 15 November 2022).
